# Identification of Key Hinge Residues Important for Nucleotide-Dependent Allostery in *E. coli* Hsp70/DnaK

**DOI:** 10.1371/journal.pcbi.1003279

**Published:** 2013-11-21

**Authors:** Peter Man-Un Ung, Andrea D. Thompson, Lyra Chang, Jason E. Gestwicki, Heather A. Carlson

**Affiliations:** 1 Department of Medicinal Chemistry, College of Pharmacy, University of Michigan, Ann Arbor, Michigan, United States of America; 2 Department of Pathology and the Life Sciences Institute, University of Michigan, Ann Arbor, Michigan, United States of America; UNC Charlotte, United States of America

## Abstract

DnaK is a molecular chaperone that has important roles in protein folding. The hydrolysis of ATP is essential to this activity, and the effects of nucleotides on the structure and function of DnaK have been extensively studied. However, the key residues that govern the conformational motions that define the apo, ATP-bound, and ADP-bound states are not entirely clear. Here, we used molecular dynamics simulations, mutagenesis, and enzymatic assays to explore the molecular basis of this process. Simulations of DnaK's nucleotide-binding domain (NBD) in the apo, ATP-bound, and ADP/P_i_-bound states suggested that each state has a distinct conformation, consistent with available biochemical and structural information. The simulations further suggested that large shearing motions between subdomains I-A and II-A dominated the conversion between these conformations. We found that several evolutionally conserved residues, especially G228 and G229, appeared to function as a hinge for these motions, because they predominantly populated two distinct states depending on whether ATP or ADP/P_i_ was bound. Consistent with the importance of these “hinge” residues, alanine point mutations caused DnaK to have reduced chaperone activities *in vitro* and *in vivo*. Together, these results clarify how sub-domain motions communicate allostery in DnaK.

## Author Summary

DnaK belongs to the highly conserved heat shock protein 70 (Hsp70) family, a group of ATP-dependent molecular chaperones that regulates proteostasis. Studies have suggested that global movements of the subdomains in the nucleotide-binding domain (NBD) of DnaK regulate its catalytic activity. However, there is less known about the key residues involved in these subdomain motions and whether these residues might also regulate inter-domain allostery with the substrate-binding domain (SBD). To examine the motions in the NBD, dynamics simulations of DnaK's NBD in the apo, ATP-bound, and ADP/P_i_-bound states were performed. Through essential dynamics and torsion angle analyses, we identified motions and highly conserved hinge residues between subdomains IIA and IIB that are likely to be important for nucleotide cycling and for communicating the nucleotide state to the SBD. Supporting this model, mutating these conserved hinge residues affected ATPase activity and chaperone functions *in vitro* and in bacteria, suggesting their importance in the nucleotide-dependent motions in DnaK.

## Introduction

*Escherichia coli* DnaK is a member of the heat shock protein 70 (Hsp70) family of molecule chaperones that assists in protein folding and minimizes protein aggregation [1]–[4]. Because of its central role in the proteostasis network, DnaK has been suggested as a promising new anti-bacterial target, and its human ortholog, Hsp70, is a drug target for the treatment of cancer [5], [6] and neurodegenerative disorders [7], [8]. These observations have led to an increased interest in understanding the structure and function of Hsp70/DnaK.

**Figure 1:**
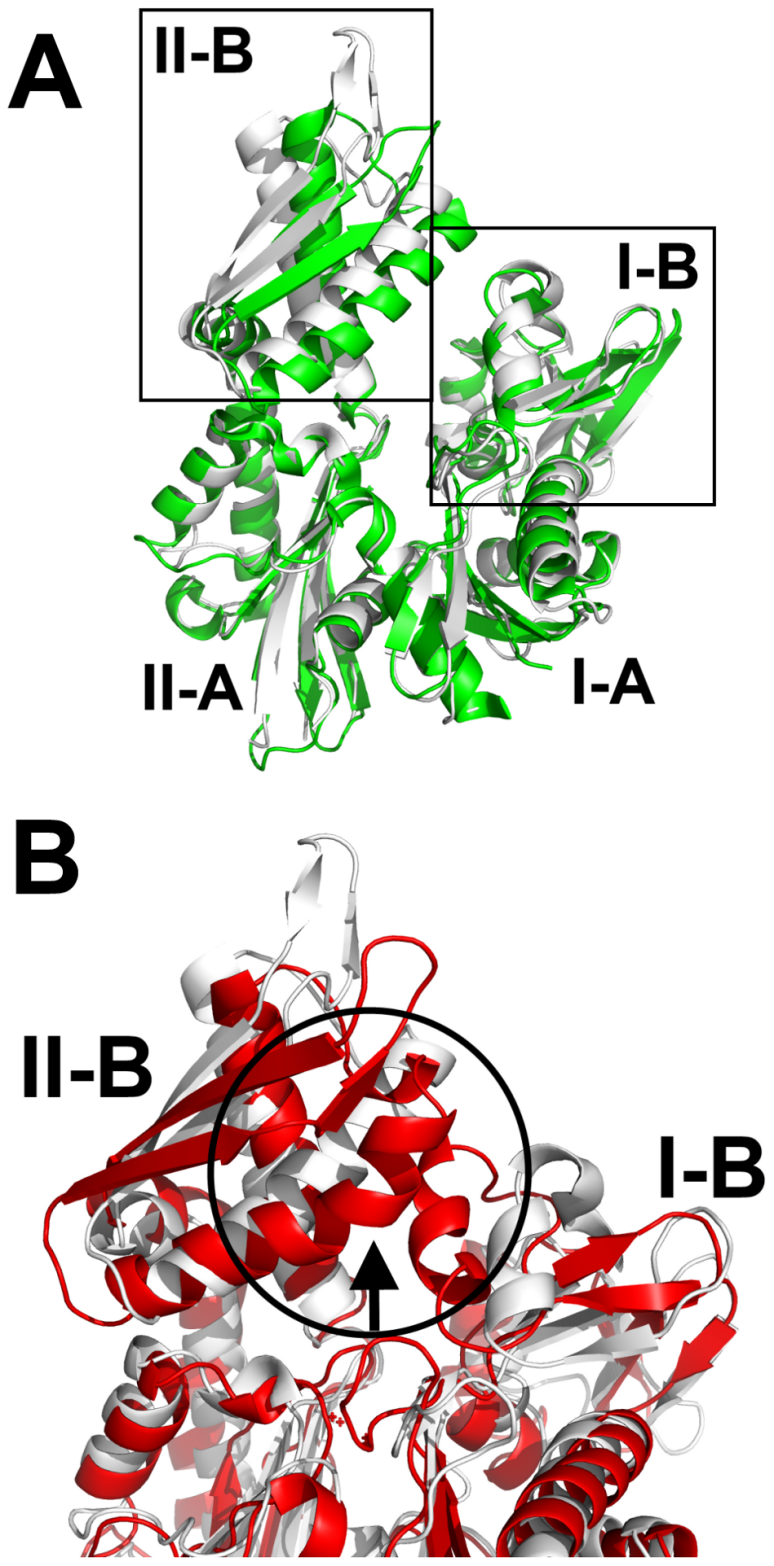
Comparison of the open and closed conformations of Hsp70/DnaK nucleotide-binding domain. (A) Light grey cartoon represents Hsc70's NBD (PDB: 1BUP) in the “closed” conformation and the green cartoon is DnaK's NBD (PDB: 1DKG) in the “open” conformation. Most of the conformational difference stems from the position of subdomain II-B relative to I-B. (B) Conformational change in the α-helix (residues 257–274) of NBD subdomain II-B. The most common conformation of the α-helix of subdomain II-B, as observed in most Hsp70 crystal structures. Bending of the α-helix near residue 262 was observed in several LD simulations (red arrow).

DnaK, like other Hsp70s, consists of a nucleotide-binding domain (NBD) and a substrate-binding domain (SBD) tethered by a flexible linker. The NBD is composed of four subdomains, I-A, II-A, I-B and II-B, arranged to form a nucleotide-binding cleft that belongs to the actin/hexokinase/Hsp70 superfamily (Figure 1A) [9], [10]. The NBD binds and hydrolyzes ATP, and this activity is important for chaperone functions. Specifically, nucleotide turnover in the NBD regulates binding of misfolded proteins in the SBD through an inter-domain allosteric network [7], [11]–[13]. The ATP-bound form of DnaK has an “open” SBD that binds loosely to misfolded proteins, while nucleotide hydrolysis in the NBD re-arranges the SBD and increases the affinity for proteins. Thus, cycling through the ATP- and ADP-bound states appears to be important in DnaK allostery and the productive refolding of denatured proteins [14].

Crystallography and NMR have been used to provide important insights into the effects of nucleotides on the structure and function of DnaK. For example, a comparison of the crystal structures of the NBD in the apo form (1DKG) [15] and the ADP-bound form (1BUP [16] and 1KAZ [17]) suggests a substantial, nucleotide-dependent movement in subdomain II-B (Figure 1A). This motion appears to involve rotations of subdomain II-B in relation to subdomain II-A, which is mediated by a sheet-coil-helix element (residues 222–234). NMR studies have further suggested that this region may act as a hinge for subdomain motions [18], [19], and more recent structural studies on an ATP-bound form of DnaK [20] further support this idea. In order to understand the specific role of the sheet-coil-helix element in this hinge motion, Chang *et al.* [21] mutated a series of residues between subdomains II-A and II-B. This work identified a number of mutations that disrupted allostery and chaperone function *in vitro* and *in vivo*. However, the underlining molecular mechanisms of these mutations are not yet clear.

Computational simulations have further advanced our knowledge of allostery in Hsp70/DnaK. For example, coarse-grained molecular dynamics of full-length Hsp70 demonstrated the collective motions that are essential in the allosteric communications between the NBD and SBD [22], [23]. Likewise, all-atom simulations identified residues essential in the binding of DnaK to nucleotide-exchange factors (NEFs) [24], and the molecular mechanism that relays the allosteric communication between the NBD and SBD [25]. Together, these studies have provided insight into the mechanism of DnaK and, more broadly, this system has contributed to our understanding of dynamics and allostery in biology.

Despite these insights, the molecular mechanisms of hinge motions in DnaK's NBD are still not clear. Specifically, it is not known which residues are essential to the hinge motions and how these molecular motions are affected by nucleotides. It is also not clear whether disrupting these hinge motions would decrease inter-domain communication between the NBD and SBD. Here, we have used dynamics simulations and mutagenesis to examine the detailed communication between the protein and the nucleotide. Conformational behavior during the simulations pointed to the allosteric importance of certain residues. These residues were then compared to their evolutionary conservation, which further highlighted the importance of the identified residues. We found that hinge residues, including G228 and its neighbors, are key regulators of this transition. They were nearly invariant across bacteria, plants, and animals, which highlights the incredible importance of these residues for chaperone function. These results provide a detailed molecular mechanism linking ATPase activity to structural transitions in DnaK/Hsp70.

## Results/Discussion

### Dynamics Simulations of DnaK's NBD

To initiate these studies, models of DnaK in different nucleotide states (apo, ATP-, and ADP/P_i_-bound) were constructed based on the crystal structure of the apo DnaK NBD (PDB: 1DKG). The nucleotide-bound models were derived from simulations of the apo state with cofactors introduced because structures of these states were not yet available at the time of this study. Since 1DKG does not carry nucleotide and metal ions, these cofactors were introduced from structures of bovine Hsc70 (PDB: 1BUP and 1KAZ). To enhance the conformational sampling, a Generalized Born, implicit-solvent model and Langevin dynamics (LD) were used for the simulations. Then, for each model, five independent trajectories were generated and confirmed to be structurally stable (Figures S1 and S2). Multiple, short trajectories were chosen to enhance sampling because it has been shown to be a more effective strategy than using a single, long-trajectory simulation [26].

When all restraints were removed, the NBD bound to either ATP or ADP/P_i_ spontaneously converted from the initial “open” conformation of an apo state to a “closed” conformation in which the subdomains I-B and II-B moved toward each other. These closed models agree well with recently reported structures that were unavailable at the start of the project. The RMSD were ∼3.2 Å and ∼4.0 Å to structures 4B9Q [27] and 4JNE [28], respectively. These values are appropriate for a very large, flexible, multi-domain protein, but the variation does point to an important limitation. To achieve the large conformational changes necessary, continuum solvent was used, and the electrostatics and salt bridges could be over stabilized, which may slightly alter the closed conformation.

Previous dynamics simulations of DnaK and its homologs bound to various nucleotides also show generally rigid motions of IIB relative to IB and IIA [22]–[25], [29]. These motions were primarily driven by movement of the entire subdomain II-B, such that the global conformation of the NBD can be described by the relative position of the intact subdomains. Thus, the distances between the center-of-mass (COM) of II-B and subdomains I-A/B were used to quantify and compare the “openness” of each conformation (Table 1). Comparing the apo, ATP-, and ADP/P_i_-bound states of NBD, we observed that the apo state adopts the most “open” conformation, with an average inter-subdomain distance of ∼32 Å. In contrast, the ATP- and ADP/P_i_-bound states adopt closed conformations, with average distances of 27.3 and 26.1 Å, respectively.

**Table 1:** Center-of-mass distance between subdomains II-B and I-A/B+II-A in Langevin Dynamics.

	Subdomain II-B to I-A/B+II-A COM Distance (Å)		
	apo	ATP	ADP/P_i_
Run 1	34.7±1.6	27.0±0.6	26.7±0.4
Run 2	29.7±0.8	27.7±0.5	25.9±0.4
Run 3	34.0 ±1.0	25.3±0.5	26.2±0.4
Run 4	27.0 ±0.5	27.9±0.4	27.1±1.6
Run 5	32.4 ±3.8	27.3±0.6	25.5±0.4
Mean	31.9 ±3.5	27.3 ±1.0	26.1±0.9

In some of the LD trajectories, the α-helix of subdomain II-B (residues 230–280) was observed to “bend” near residues 262–267 (Figure 1B). This bending did not appear to be dependent upon the nucleotide state because it was observed in the simulations of the apo, ATP-, and ADP/P_i_-bound NBDs. Similar helix bending has also been described in actin and a phosphatase with a similar fold [9]. Although the functional importance of this transition is not clear, it may be involved in assisting with the full opening and closing of the NBD.

### Essential Dynamics

**Figure 2:**
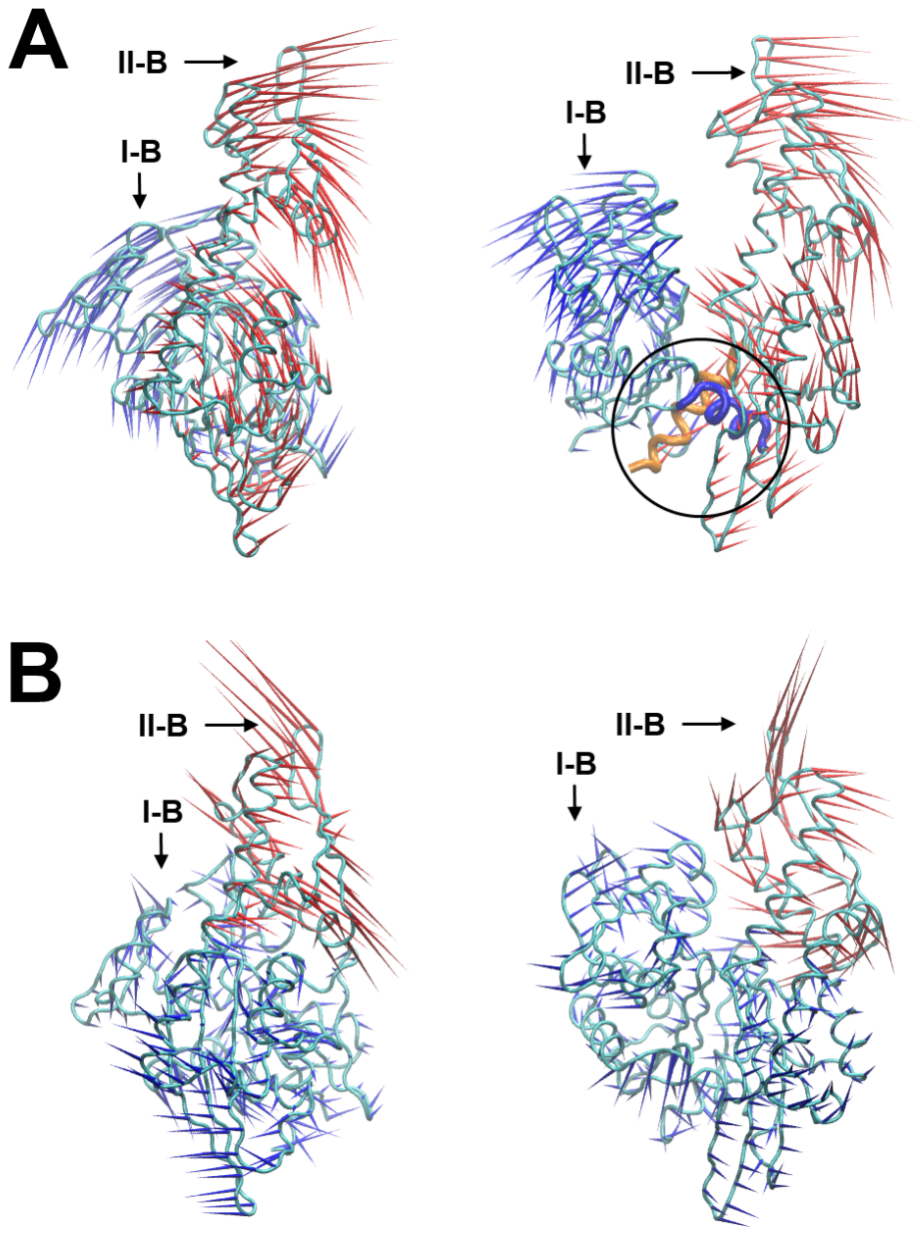
Eigenvectors of NBD. (A) The first eigenvector of NBD essential dynamics (ED) involves a shearing motion of the domains I and II. *Left* – side view of the NBD, in which ED vectors of domains I and II are colored blue and red, respectively. *Right* – front view of the NBD. The shearing motion of domains I and II is manifested through the two helices at the interface of subdomains I-A and II-A, as indicated in the circle. (B) Second eigenvector of NBD ED involves a rotating motion of primarily subdomain II-B, which may change the distance between subdomains I-B and II-B. Subdomain II-B is colored red, and the other three subdomains are in blue.

To study conformational changes in the NBD, essential dynamics (ED) was used to examine the LD trajectories of each model. Previous studies have shown that combinations of several independent dynamics simulations often provide sufficient sampling of conformational space for ED [30]. Thus, five independent LD trajectories were combined for each model and analyzed with ED. In all cases, the first 5 eigenvectors (first – fifth) represented ∼80% of the principle motions of the protein (Figure S3). The apo and nucleotide-bound models yielded generally similar global motions, and the lowest mode of the ED was a shear motion between subdomains I and II (Figure 2A). This motion involved two crossed α-helices (171–179 and 367–377) with inter-helical angles of approximately 70° to 80°. This shear motion occurred in the interface between subdomains I-A and II-A, and it manifested as a large displacement between subdomains I-B and II-B. Actin and glucokinase, which both have similar folds to DnaK, have been shown to undergo a similar shear motion [31], [32]. The second eigenvector involved a rotational motion of subdomain II-B, in which II-B moves relatively independent to the other subdomains (Figure 2B). This motion appeared to be a hinged-motion between subdomains II-A and II-B. Together, the first and second eigenvectors described motions involved in the opening and closing of the nucleotide-binding site, and these motions are consistent with experimental observations from NMR [19].

### Torsion Angle Analysis

The movement of protein domains is often linked to protein function [33], and these movements typically involve the motion of rigid domains relative to each other [31], [34]. These structural changes cluster into two major mechanisms: shear movements in which domains slide along one another while maintaining closely packed interfaces, and hinged movements in which relatively disordered regions connecting the domains undergo significant backbone conformational changes [31]. Hinged movements usually involve a small set of residues that adopt distinct torsion angles of their φ (C′-N-C^α^-C′) and ψ (N-C^α^-C′-N) bonds, enabling these individual “hinge” amino acids to mediate larger motions between domains or subdomains [34]. Therefore, it is often possible to identify the key hinge residues by examining which amino acids undergo changes in their φ-ψ torsion angles during dynamic simulations.

**Figure 3:**
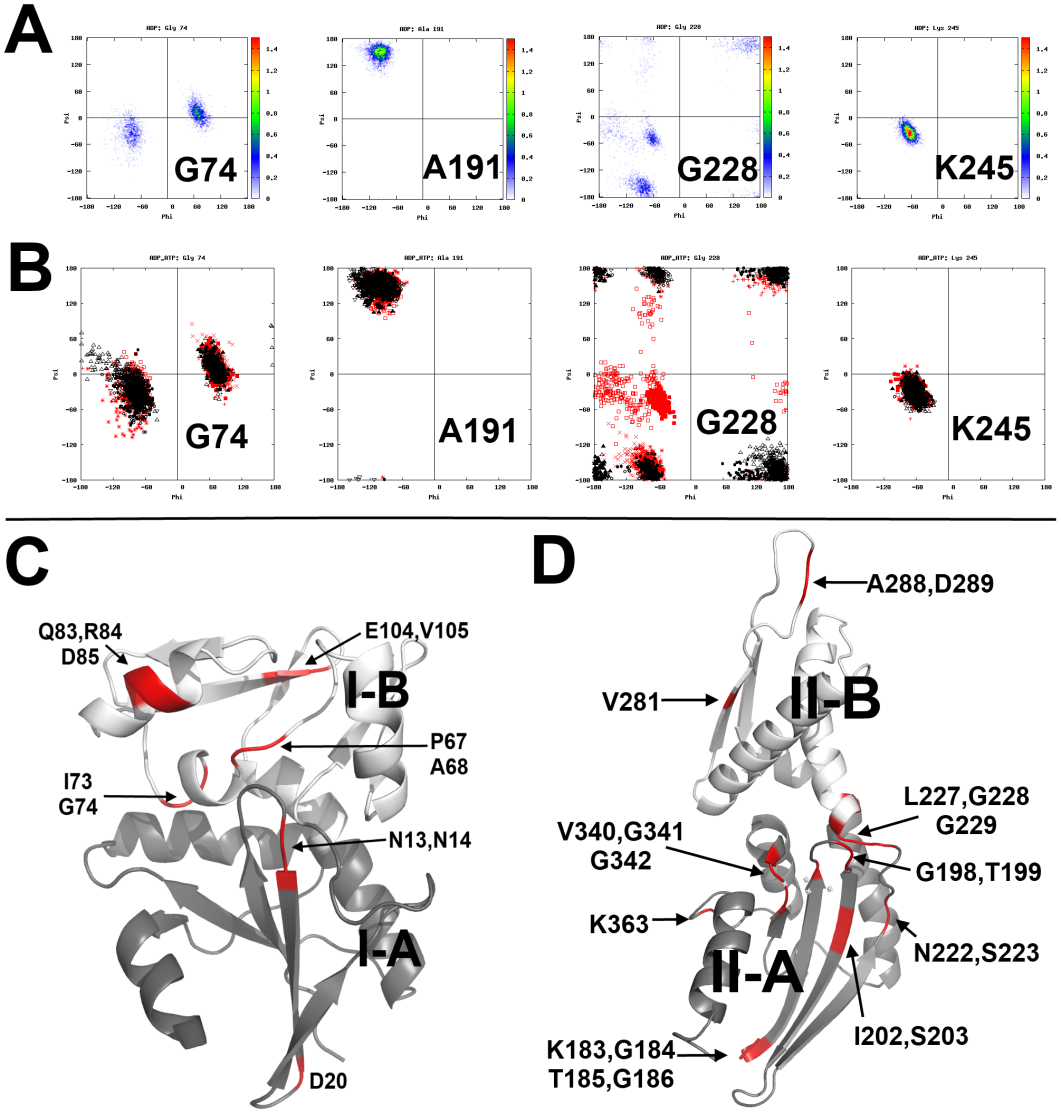
Examples of Ramachandran plots of NBD residues in the LD simulations. (A) Density maps of the φ-ψ torsion angles that were highly occupied (red). Residues not involved in hinged movement usually had one φ-ψ torsion angle cluster in the plot (A191 and K245). However, other “hinge” residues had two or more φ-ψ clusters (G74 and G228), which indicated substantial conformational changes in the backbone. (B) Comparing the ψ-φ clusters generated from the ADP/P_i_-bound (red) and ATP-bound (black) trajectories. Residues not involved in hinged movement (A191, K245) have overlapped φ-ψ clusters, while hinge residues have φ-ψ clusters that differ significantly. Some of these hinge residues were affected by nucleotide changes (G228), while others were not (G74). (C) Cartoon representation of Domain I, in which residues have multiple Cα φ-ψ states during dynamics simulations are shown. Subdomain I-A is colored in gray and I-B in white. (D) Cartoon representation of Domain II. Subdomain II-A is colored in gray and II-B in white. Residues with multiple φ-ψ states are colored in red. Most colored residues are located in random coils, loops or end of the α-helices or β-sheets.

One of the key questions in the DnaK system is which residues might be involved in the nucleotide-dependent hinge motions. To detect such hinge residues, we performed C_α_ torsion angle analysis on the NBD trajectories (Figure 3A and 3B). Our Ramachandran results of the amino acids are similar to those described by Hovmöller *et al.* [35], in which residues in the helix, sheet, and random coil region have different φ-ψ distributions. As expected, we found that the great majority of residues, such as A191 and K245, do not have the properties of hinged movement, such that the Ramachandran plot of these residues show a single φ-ψ torsion angle cluster throughout the simulations. In contrast, a small number of residues, such as G74, had more than one conformation evident from multiple φ-ψ clusters, which suggests that they may be involved in hinged movement (Table 2). Further, a subset of these residues (N13, N14, P67, A68, I73, R84, D85, T184, T185, G223, L227, G228, and G229) had multiple conformations with distinct φ-ψ clusters. Further, some of these φ-ψ clusters were correlated with changes in nucleotide state.

**Table 2:** Residues with multiple φ-ψ clusters in dynamic simulations.

	Residue
I-A (1–36, 111–180)	N13, S14, D20
I-B (37–110)	G51, P67, A68, I73, G74, Q83, R84, D85, E104, V105
II-A (181–226, 311–383)	K183, G184, T185, G186, L195, G198, T199, I202, S203, N222, G223, L227, G228, G229, V340, G341, G342, K363
II-B (230–310)	V281, A288, D289

Next, we examined the location of the residues with multiple φ-ψ clusters on the DnaK NBD structure. These residues are found in all 4 subdomains with a bias for the flexible loops between subdomains I-A and I-B (Figure 3C). Additionally, several clusters were grouped in subdomain II-A, including residues 183–186 and 340–342, which form flexible loops, and residues 195, 198 and 199, which are found in a flexible β-hairpin that coordinates with the phosphates of the nucleotide (Figure 3D).

In DnaK, the lobe between I73/G74 and E104/V105 consists of a helix-loop-sheet structure. We found that this lobe is capped by two sets of residues with multiple φ-ψ clusters (Table 2 and Figure 3C), suggesting that these residues might function as hinges. This movement might be interesting because this lobe is directly adjacent to the nucleotide-interacting residue R71, which plays a critical role in enzymatic activity.

**Figure 4:**
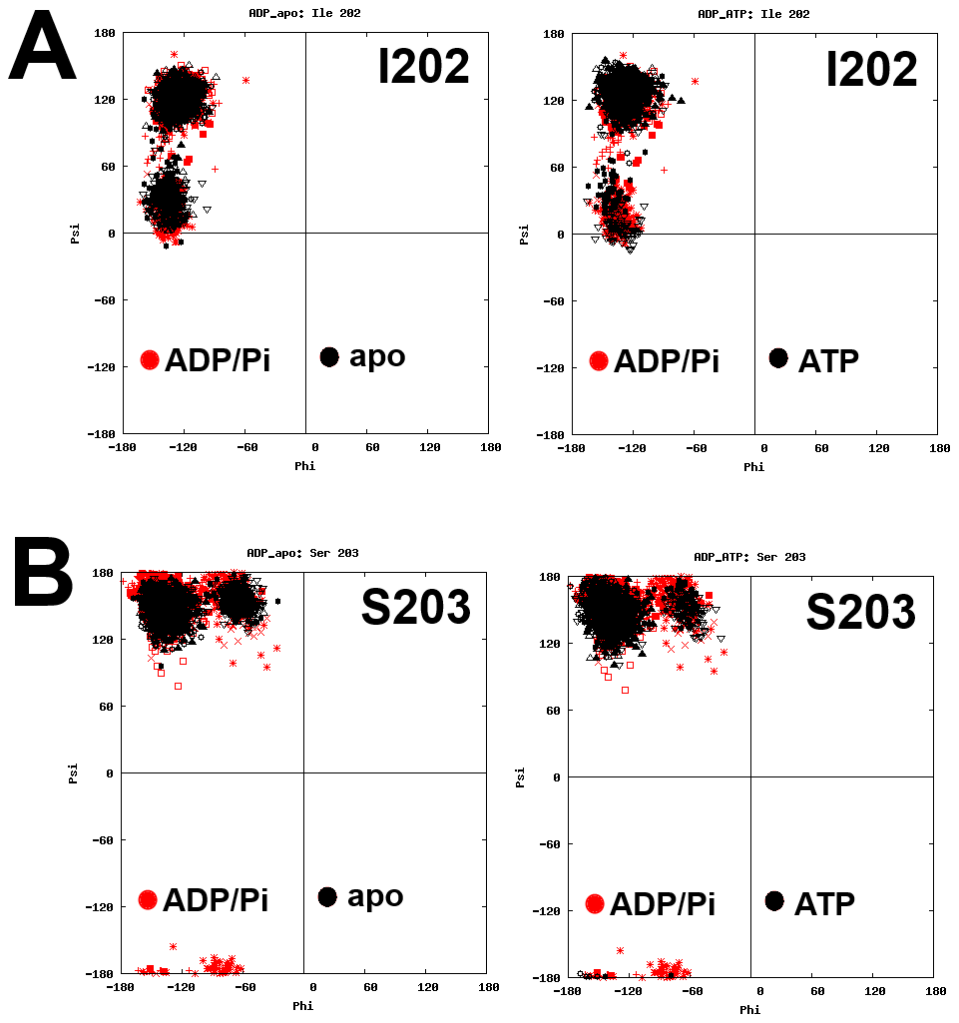
Ramachandran plots of NBD residues in different nucleotide-bound states. (A) I202 and (B) S203. The φ-ψ torsion angle clusters do not appear to respond to change in nucleotide-bound state.

**Figure 5:**
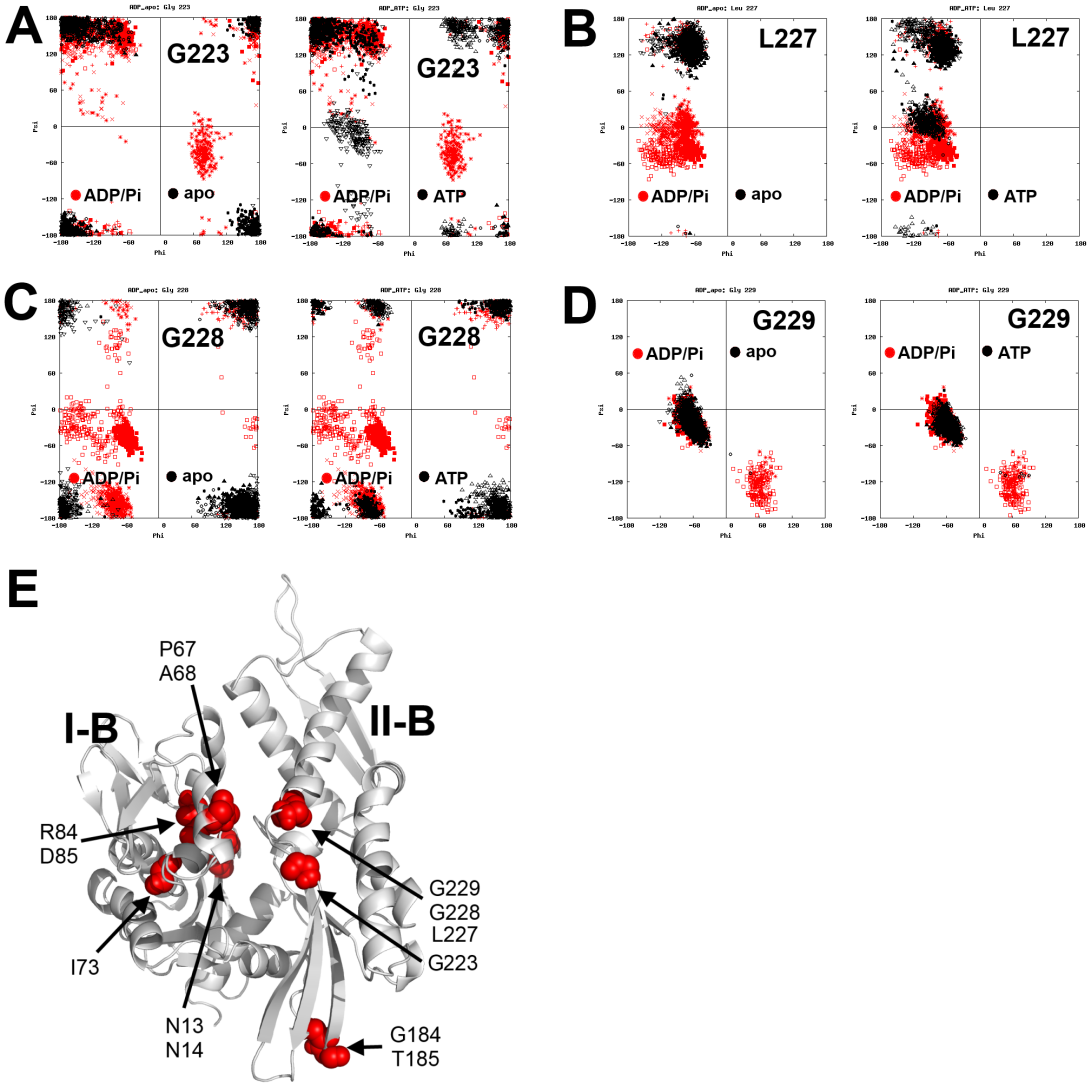
Ramachandran plots of residues in different nucleotide-bound states. (A) G223, (B) L227, (C) G228, and (D) G229. These residues had clusters of φ-ψ torsion angles that were significantly different in the ADP/P_i_ state compared to the apo state and ATP-bound state. (E) The locations of these nucleotide-sensitive residues are shown in red.

Finally, another significant cluster of residues with multiple φ-ψ clusters was identified near the junction between the subdomains II-A and II-B. These clusters included residues in β-sheets (I202, S203) and random coils (G223, L227, G228, G229). The β-sheet residues were not sensitive to nucleotide state (Figure 4), implying that they may be involved in intrinsic protein dynamics. However, residues G223, L227, G228, and G229 had multiple φ-ψ states, and their states changed in response to nucleotide (Figures 5A–E). These residues seemed like good candidates for playing a key role in nucleotide-dependent structural transitions and allostery, as suggested by Chiappori *et al.* [25].

### Correlations to Nucleotide State

As a complementary way to understand how nucleotides might affect motions in DnaK, the correlation matrices of each trajectory from the LD simulations were compared. Then, residues with motions strongly correlated with ATP or ADP were extracted and ranked according to their degree of absolute correlation (1.0 correlation was just as important as −1.0 anti-correlation). The 60 most-correlated residues were tallied for each LD trajectory. Residues that were highly correlated in at least 4 of the 10 independent trajectories were considered significant (Table 3).

**Table 3:** Residues with strong correlation to nucleotide in NBD LD simulations.

Subdomain	Residue
I-A	D8, C15, D33, P37, S38, P143, A144, Y145
I-B	I39, G51, L66, A68, L72
II-A	D194, L195, F200, G223, T225, G228, G229, V230, L339, T344, M346, P347, V349
II-B	A265, K268, A269, S274

**Figure 6:**
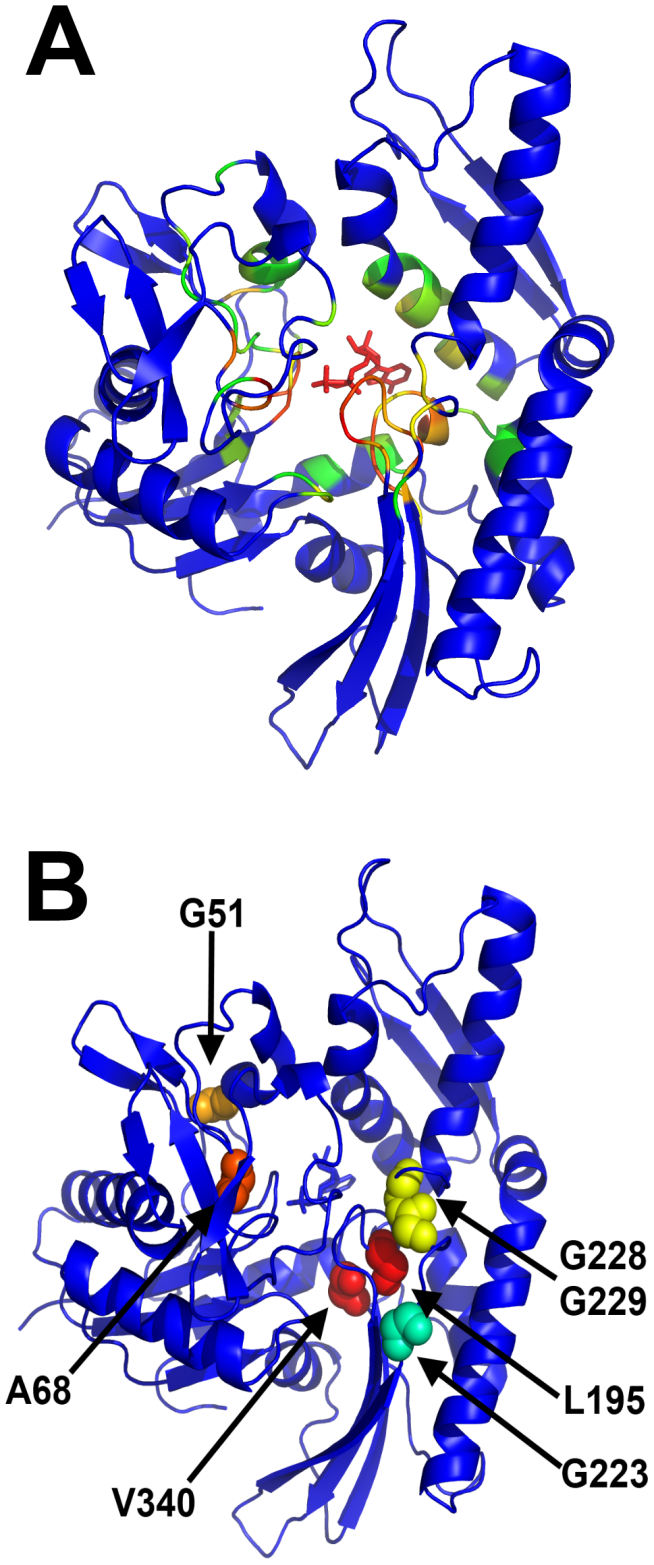
The motions of the nucleotides are correlated with several residues. (A) Residues correlated with the nucleotides, listed in Table 3, are colored by how frequently the behavior is observed in the LD simulations of ATP- and ADP/P_i_-bound NBD: red, 10 out of 10; yellow, 6 out of 10; green, 4 out of 10; blue <4. (B) Most of the correlated residues are in the nucleotide binding site; here, we highlight the residues with multiple φ-ψ torsion angle clusters, strong correlated motion to nucleotides, and >8 Å away from nucleotide.

Residues with weak or no correlation to the nucleotides were largely found on the surface of the NBD and at the flexible loops at the distal ends of the subdomains. A number of residues that have multiple φ-ψ states, such as P67, E104, and A288, do not have strong correlation to the nucleotides and were not considered for further study. In contrast, residues with strong correlation were found in the interior of all four subdomains. As expected, most of these residues were in the vicinity of the nucleotide-binding site (<8 Å from the nucleotide), but a few were further away (Figure 6A). Comparing these residues to those identified in the φ-ψ torsion angle analysis, we found that several residues are present in both lists, including G51, A68, L195, G223, G228, G229, and V340 (Figure 6B).

An examination of these residues suggested that they may have significant effects on the functionality of NBD. For example, A68 is adjacent to K70 and R71, key residues that bind to nucleotides [36]. Likewise, L195 and V340 may interact with I202 and S203 to define the shape of the nucleotide-binding site. Residues G223, G228, and G229, were especially interesting because (as mentioned above) they are located away from the nucleotide-binding site and situate in a random coil that tethers subdomains II-A to II-B.

To further explore the potential importance of the “hinge” residues, we studied which residues had correlated motions with G223, G228 and G229. Dynamics simulations of NBD in the apo, ATP-bound, and ADP/P_i_-bound states were examined, and among the top 60 strongly correlated and anti-correlated residues, we found strong enrichment of components of the known proline switch that consists of residues K70, R71, P143, A144, Y145, F146, R151 and E171. The proline switch has previously been found to regulate allostery in Hsp70 chaperones [36]. In simulations of the NBD in all the apo-, ATP- and ADP/P_i_-bound states, K70 and R71 were consistently correlated with each of the “hinge” residues (i.e., G223, G228, and G229). In contrast, P143, A144, Y145, F146, R151, and E171 correlated to G228 more frequently in the ATP-bound and ADP/P_i_-bound states than in the apo-state. This result might be expected because NBD in the apo-state simulations had a wider range of movements (Figure S1). In all cases, correlations between the hinge residues and the proline-switch residues appeared more frequent in the ATP-bound state than in the ADP/P_i_-bound state. These results suggest that interactions among ADP, P_i_, the hinge residues, and the proline-switch residues may be looser than when ATP is bound.

### Hinge Residues Are Highly Conserved

To understand whether the proposed φ-ψ “hinge” residues might be preferentially conserved among Hsp70 family members, BLASTP [37] was used to align the sequences of DnaK and all other available members of the Hsp70 family (3700 orthologs) from the GenBank database. Several residues in the random coil that tether subdomains II-A and II-B (G223, L227, G228, and G229) were highly conserved among bacteria, with over 90% identity and nearly 100% similarity. Remarkably, *high conservation was also observed across all kingdoms, particularly for L227, G228, and G229* (Table 4). This level of conservation was greater than expected for the average residue in these chaperones; the overall conservation between the prokaryotic *E. coli* DnaK and the eukaryotic human Hsp70 is ∼50%. These findings suggest that the highly conserved residues might be especially important for the function of Hsp70 family members.

**Table 4:** Conserved residues in subdomains II-A/II-B hinge region of DnaK NBD.

	Identity (*Conserved mutation*)		
Residue	Bacteria (1714 Orthologs)	Animals (1388 Orthologs)	Plants (597 Orthologs)
I202	31% *(64%)*	5% *(90%)*	5% *(83%)*
**S203**	**90% (** ***9%*** **)**	**92% (** ***7%*** **)**	86% (*10%*)
E217	77% (*16%*)	87% (*9%*)	79% (*12%*)
A220	55% *(41%)*	48% *(51%)*	59% *(39%)*
T221	83% *(13%)*	**94% ** ***(4%)***	88% *(5%)*
N222	41% *(41%)*	14% *(11%)*	17% *(22%)*
**G223**	**98% (** ***1%*** **)**	**91% (** ***1%*** **)**	83% (*1%*)
D224	83% *(11%)*	**94% ** ***(4%)***	**91% ** ***(5%)***
T225	50% *(41%)*	89% *(2%)*	82% *(3%)*
H226	36% *(18%)*	83% *(4%)*	82% *(4%)*
**L227**	**97% (** ***2%*** **)**	**96% (** ***3%*** **)**	**92% (** ***7%*** **)**
**G228**	**99% (** ***1%*** **)**	**99% (** ***0%*** **)**	**98% (** ***1%*** **)**
**G229**	**100% ** ***(0%)***	**99% ** ***(0%)***	**99% ** ***(1%)***
E230	30% *(64%)*	87% *(8%)*	68% *(27%)*

Identity above 90% are highlighted.

### Experimental Testing of DnaK Point Mutations – Residue G228 Is Critical for Chaperone Functions

Next, we designed mutations of residues in DnaK to understand their potential role in allostery and chaperone functions. In this effort, we focused on residues that met two criteria: those that were highly conserved (>90% identity in bacteria and animals, Table 4) and those that were also predicted by the simulations to be involved in φ-ψ hinge motions (see Table 2 and Figures 4 and 5). The resulting five residues (Table 5) were placed into two groups, depending on whether their motions were correlated with nucleotide.

**Table 5:** Activity of DnaK and its mutants.

	ATPase Activity (µM P_i_/µg DnaK/hr)	Fold Change	Luciferase Refolding Activity (%)	Bacterial Growth (% of WT)^a^
WT	11.3±0.2	–	100±12	–
	*Hinge Residues Not Correlated to Nucleotide Motion*			
I202A	7.4±0.6	0.7	77±10	75
S203A	20.8±0.2	1.8	101±12	110
L227A	8.4±0.9	0.7	80±14	25
	*Hinge Residues Correlated to Nucleotide Motion*			
G223A	11.5±0.2	1.0	81±14	22
G228A	18.5±0.6	1.6	12±3	13

a ref. [21].

We then expressed and purified wild type DnaK and a series of DnaK mutants in which each of the proposed hinge residues were replaced with an alanine [21]. All of the purified proteins were properly folded as determined by thermal stability and circular dichroism measurements (Figure S4), suggesting that the alanine mutations did not damage the overall fold or stability of the chaperone.

To assess the effects of the mutations on function, we measured the ATPase activity of the mutants using a malachite-green assay [38]. We also measured the ability of the chaperone to refold denatured firefly luciferase, which is an assay that is commonly used as a measure of chaperone functions *in vitro* [39]. Changes in the ATPase activity of DnaK are expected to impact luciferase refolding because this process requires multiple cycles of binding between DnaK and the denatured luciferase [14]. Thus, ATP cycling that is either too fast or too slow might imbalance the dwell time of DnaK on luciferase and decrease overall folding efficiency [21]. Further, extensive studies have revealed that the NBD and SBD of DnaK communicate through inter-domain interactions [18], [20], [40]. Thus, if the putative hinge residues are important in controlling NBD allostery, mutations in these positions would be expected to disrupt normal ATP turnover and, in turn, decrease chaperone functions, such as protein refolding, both *in vitro* and *in vivo*. Importantly, this system appears to be highly sensitive to even modest changes in ATPase activity. For example, chemical compounds that impact steady-state ATP turnover by only 20–50% [41], [42] or single turnover by only about 20% [43], [44] have profound effects on Hsp70/DnaK biology in cellular assays measuring the stability of chaperone protein substrates.

These experiments revealed that mutations I202A and L227A decreased ATPase activity by ∼30% and refolding function by ∼20%, suggesting that these highly conserved residues are important for allostery in DnaK. Mutation G223A had no effect on ATPase activity, but it did have a modest effect on refolding activity (∼80% of wild type). The S203A mutant had normal refolding activity, but it had accelerated ATPase function (∼180% of wild type).

Finally, G228A had dramatically enhanced ATPase activity (∼160% of wild-type), yet it had only 16% of the wild-type refolding activity. The results with G228A were particularly striking, supporting the idea that G228 is an important hinge residue. G228 is located between subdomain II-A and II-B, and it likely dictates the relative movement of the two subdomains, as observed in the second mode of the essential dynamics of NBD (see Figure 2B). It is situated between residue L227 and G228, which also show altered φ-ψ behavior based on the nucleotide-bound state in Figure 5. These experimental results confirmed the importance of the residues identified in the LD simulations.

To test whether these mutations might disrupt the physiological functions of DnaK, we next expressed the hinge mutants in *E. coli* (DE3) cells that lack endogenous chaperone (Δ*dnak*) [21]. DnaK is normally required for growth of bacteria at elevated temperatures (∼37°C), so we measured their growth under stress conditions to assess whether the point mutant could recover wild-type DnaK activities. Compared to cells in which WT DnaK was restored, we found that the mutations that damaged luciferase-refolding activity (I202A, L227A, G223A, and G228A) had a significantly reduced ability to recover bacterial viability (Table 5; growth was 75%, 25%, 22%, and 13% of WT, respectively). Conversely, the S203A DnaK mutant, which had little effect on luciferase refolding, had nearly normal viability (110% of WT). These results support the importance of the hinge residues, especially G228, on DnaK functions. These results also support a model in which luciferase refolding activity is correlated with the global, *in vivo* functions of DnaK [21].

Nucleotide-dependent structural transitions in DnaK are linked to its chaperone functions. While there is much known about the allostery between the NBD and SBD in DnaK [18], [22], [45], there is less known about the key residues involved in the subdomain motions of the NBD and how they impact NBD-SBD communication. Using dynamics simulations of DnaK's NBD in the apo, ATP-, and ADP/P_i_-bound states, we identified shear motions between subdomains I-A and II-A and a hinge motion that is consistent with recent reports [19], [20]. Together, these motions result in a dramatic movements of the nucleotide-binding cleft, which is likely important for nucleotide cycling and for communicating the nucleotide state to the SBD. Using the results of these simulations, we further identified residues, including G223, L227, G228, and G229, as being potentially important to the hinge motions. These residues are very highly conserved through evolution, and they are located at what appeared to be a critical hinge region. These results were supported by torsion angle analyses, which suggested that these residues adopt two distinct backbone conformations. For some of these residues, these clusters correlated with nucleotide state. Finally, mutation of these residues largely supported this model and showed that chaperone functions were misregulated when the hinge residues were mutated to alanine. Together, these studies point to specific hinge residues that are nearly invariant across all kingdoms of life and are important for the nucleotide-dependent motions in DnaK. These findings are important for understanding structure and function in DnaK.

## Materials and Methods

### Molecular Dynamics Simulations

Coordinates of *E. coli* DnaK NBD in complex with the nucleotide-exchange factor, GrpE (PDB: 1DKG [15]) were obtained from the PDB, and GrpE was discarded. The missing side chains and short loops of NBD were introduced using Molecular Operating Environment [46] version 2005.06. AMBER 10 [47] was then used to perform unrestrained all-atom LD simulations. Models of DnaK NBD in different states (apo-, ATP-, and ADP/P_i_-bound) were built for these simulations. The structure 1DKG was crystallized without bound cofactors, so the essential ions, ATP, and ADP/P_i_ were introduced by transferring the coordinates from the crystal structures of the closely related homolog, bovine Hsc70. The cofactors ADP, PO_4_^3−^, Mg^2+^, and K^+^ were obtained from the 1BUP [16] structure whereas ATP, Mg^2+^, and K^+^ ions were obtained from 1KAZ [17]. Note that the ions are not explicit counter ions; they are bound to the protein and play a structural role.

The protein was modeled using the FF99SB force field [48]. Parameters for P_i_ and Mg^2+^ were generated using the ANTECHAMBER module of AMBER with GAFF [49], [50] and AM1-BCC charges [51], [52]. Parameters developed by Meagher *et al.* [53] were used for ATP and ADP. The SHAKE algorithm [54] was used to restrain hydrogen atoms. Model II (igb = 5) of a modified Generalized Born approach [55] was used to implicitly model aqueous solvation. Collision frequency of 1 ps^−1^ was used. A non-bonded interaction cutoff of 999 Å was used. Default dielectric values were used: interior = 1 and exterior = 78.5.

Five independent LD simulations for each of the NBD states (apo, ADP/P_i_-bound, and ATP-bound) were initiated with different random-number seeds. Hydrogen atoms were first minimized, followed by residue side chains, and finally an all-atom minimization. Heating and restrained equilibrations were followed, in which the system was heated gradually from 100 to 300 K during the first two equilibration phases, and the temperature remained at 300 K for the remaining equilibration and production phases. Restraints were placed on all heavy atoms and gradually relaxed over the first four equilibration phases, using force constants from 2.0 to 0.1 kcal/mol·Å^2^. Restraints were maintained on backbone atoms in the fifth equilibration phase, using a force constant of 0.1 kcal/mol·Å^2^. All restraints were removed in the sixth phase. The first three phases were performed for 20 ps each, followed by 50 ps for the fourth and fifth. The sixth phase, unrestrained equilibration, was run for 740 ps, and the production phase was run for 5 ns. A time step of 1 fs was used, and snapshots were collected every 1 ps. Results of the LD simulations were analyzed by calculating the variance-covariance matrix of the trajectory using the ptraj module of AMBER. In-house scripts were used to parse subsets of data and to plot the correlation results for visualization and analysis.

### Protein Purification

The *E. coli dnaK* genes were amplified by PCR using Platinum *Pfx* DNA polymerase and inserted into the pMCSG7 plasmid as Ndel-HindIII fragment through ligation-independent cloning [56]. The primers for site-directed mutagenesis (see the Supplemental Information) were designed based on the report of Zheng *et al.* [57], and QuikChange mutagenesis was carried out following the manufacturer protocol. Based on the simulations, the following *dnaK* mutants were made: I202A, S203A, G223A, L227A, and G228A. Each mutant and the wild-type (WT) DnaK was expressed as an N-terminal His-tagged protein and purified from BL21(DE3) cells as previously described [21], [38]. Purity of the proteins was greater than 90%, as judged by Coomassie staining. Circular dichroism spectra were consistent with the structure of folded NBD. Proteins were frozen on liquid nitrogen and stored at −80°C until use.

### Thermal Stability Assay

ThermoFluor is a fluorescence-based thermal shift assay system that assesses protein stability [58]–[60]. To test the relative stability of DnaK and its mutants, each protein was diluted to 0.2 mg/mL in either buffer A (100 mM sodium phosphate, 5 mM MgCl_2_, 20 mM KCl, pH 7.2) or buffer B (100 mM HEPES, 5 mM MgCl_2_, 20 mM KCl, pH 7.2), as indicated. To these solutions was added ADP or ATP (1 mM) and 1,8-ANS (100 µM). These samples were placed in 384 well plates and covered with silicone oil to minimize evaporation. The fluorescence of ANS was monitored with excitation/emission at 375/480 nm. Measurements were performed in continuous mode, using a temperature range of 30 to 85°C and increments of 1°C.

### ATPase, Refolding, and Bacterial Growth Assays

ATPase activity, firefly luciferase refolding assays, and bacterial growth studies were carried out as previously described [21]. Briefly, steady-state phosphate release from ATP was measured using a malachite green assay. Chaperone-dependent refolding was measured by Steady Glo after incubation of chemically denatured firefly luciferase with DnaK (or DnaK mutants), DnaJ and ATP. For the bacterial growth assays, the DnaK point mutants were expressed under control of a T7 promoter (pMCSG7) in *E. coli* Δ*dnak* (DE3) cells. Mutant strains expressed approximately equal amounts of either WT or mutant DnaK, as judged by Coomassie stains. The growth of bacteria expressing the mutants was assessed by measuring optical density (OD_600_) at 37°C for 4 hours. Results are reported as a percentage of the growth of Δ*dnaK* cells in which WT DnaK was restored from the pMCSG7 vector.

## Supporting Information

Figure S1 Stability of DnaK NBD in LD simulations. Three nucleotide-bound states were modeled. In the production run (after 0.75-ns), apo state simulations remained in the “open” conformation, with large fluctuations in C_α_ RMSD, ∼4.9±1.4 Å. The NBD in either ADP- or ATP-bound states converted from an initially “open” conformation to a “closed” conformation, leading to a relatively high Cα RMSD. The mean Cα RMSD of ADP-DnaK and ATP-DnaK complexes were ∼4.6±0.8 Å and ∼4.1±0.6 Å, respectively. However, the trajectories became stable once closing occurred.
(TIF)

Figure S2 Stability of cofactors in the LD simulations. The ADP-bound NBD complex contained both ADP and P_i_. In the production run (after 0.75-ns), the mean heavy-atom RMSD of ADP and P_i_ in the ADP-DnaK NBD complex were 4.7±0.9 Å and 6.4±1.1 Å, respectively. For ATP in the ATP-DnaK NBD complex, the mean heavy-atom RMSD throughout the trajectories was 3.7±0.7 Å.
(TIF)

Figure S3 Normalized cumulative eigenvalues of NBD essential dynamics. In all cases, 25 eigenvectors describe >90% of the essential motions in the simulations, while 5 of the lowest modes are sufficient to describe ∼80% of the essential motions (vertical line).
(TIF)

Figure S4 Normalized circular dichroism spectra of wild-type and mutant DnaK. All mutants have very similar fold as the wild-type DnaK.
(TIF)

Text S1 Primers for DnaK mutagenesis. The detailed genetic codes used for the DnaK mutants are given.
(DOCX)
