# Supplementary figures and images for: Identification of Key Hinge Residues Important for Nucleotide-Dependent Allostery in E. coli Hsp70/DnaK
Source: PLoS Comput Biol. 2013 Nov 21;9(11):e1003279. doi: 10.1371/journal.pcbi.1003279 (PMC3836694; doi:10.1371/journal.pcbi.1003279)

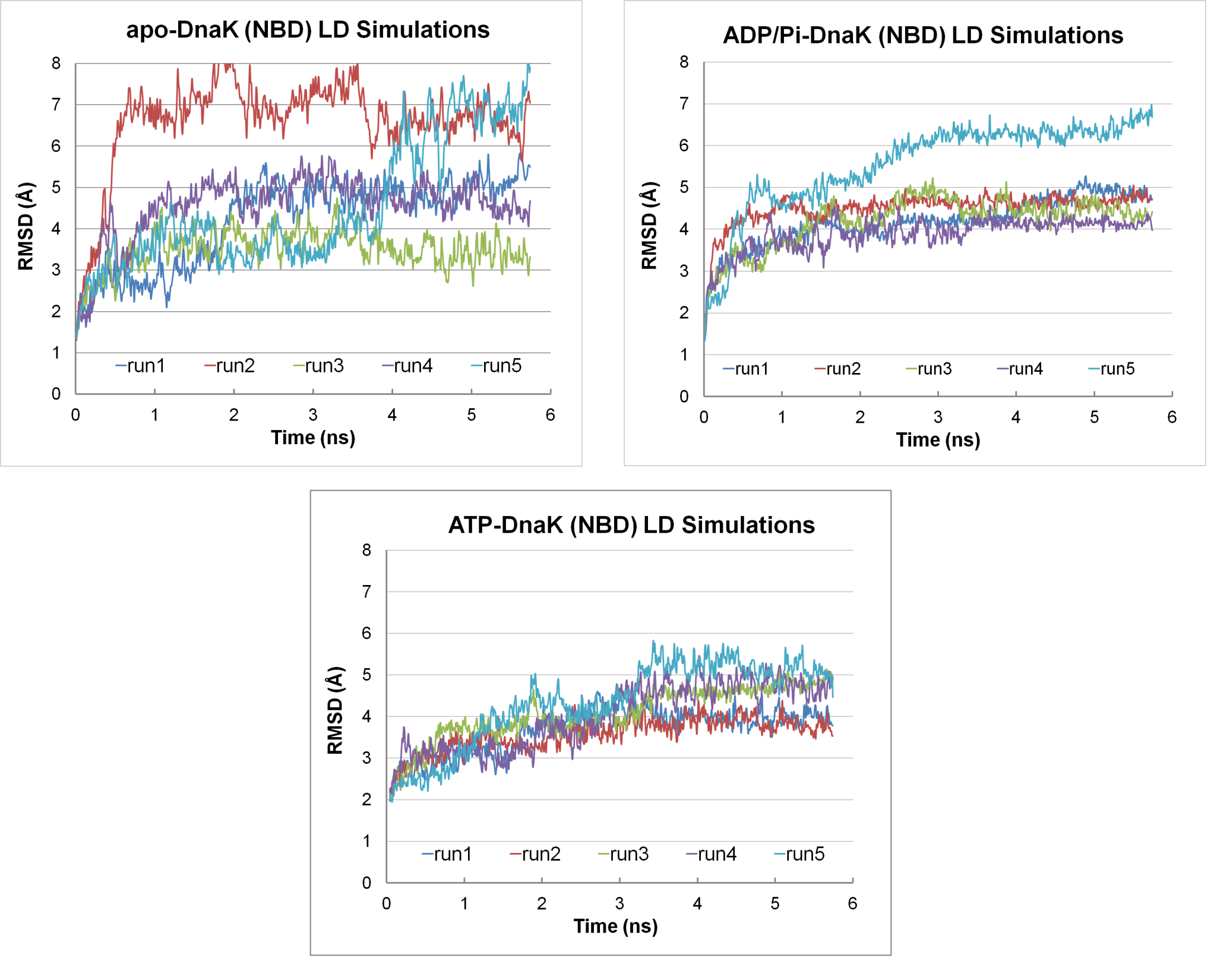

Supplement: Figure S1 — Stability of DnaK NBD in LD simulations. Three nucleotide-bound states were modeled. In the production run (after 0.75-ns), apo state simulations remained in the “open” conformation, with large fluctuations in Cα RMSD, ∼4.9±1.4 Å. The NBD in either ADP- or ATP-bound states converted from an initially “open” conformation to a “closed” conformation, leading to a relatively high Cα RMSD. The mean Cα RMSD of ADP-DnaK and ATP-DnaK complexes were ∼4.6±0.8 Å and ∼4.1±0.6 Å, respectively. However, the trajectories became stable once closing occurred. (TIF) [file pcbi.1003279.s001.tif]

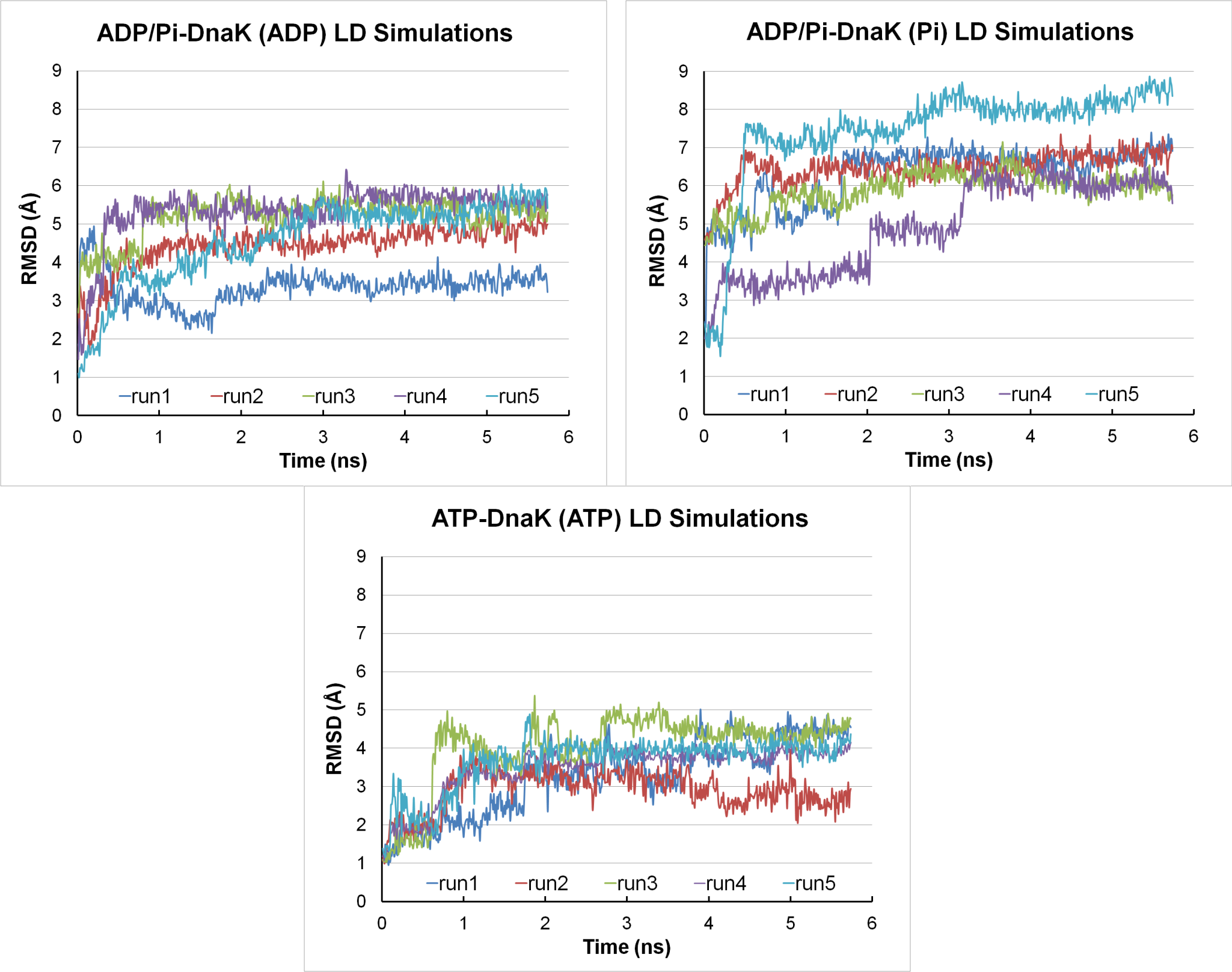

Supplement: Figure S2 — Stability of cofactors in the LD simulations. The ADP-bound NBD complex contained both ADP and Pi. In the production run (after 0.75-ns), the mean heavy-atom RMSD of ADP and Pi in the ADP-DnaK NBD complex were 4.7±0.9 Å and 6.4±1.1 Å, respectively. For ATP in the ATP-DnaK NBD complex, the mean heavy-atom RMSD throughout the trajectories was 3.7±0.7 Å. (TIF) [file pcbi.1003279.s002.tif]

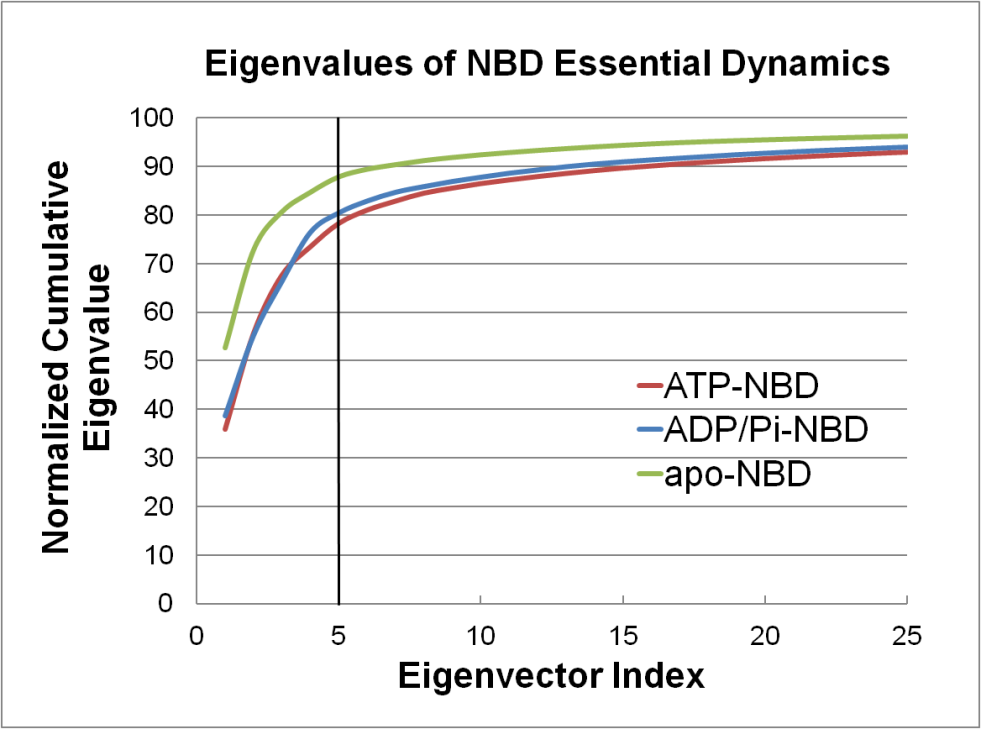

Supplement: Figure S3 — Normalized cumulative eigenvalues of NBD essential dynamics. In all cases, 25 eigenvectors describe >90% of the essential motions in the simulations, while 5 of the lowest modes are sufficient to describe ∼80% of the essential motions (vertical line). (TIF) [file pcbi.1003279.s003.tif]

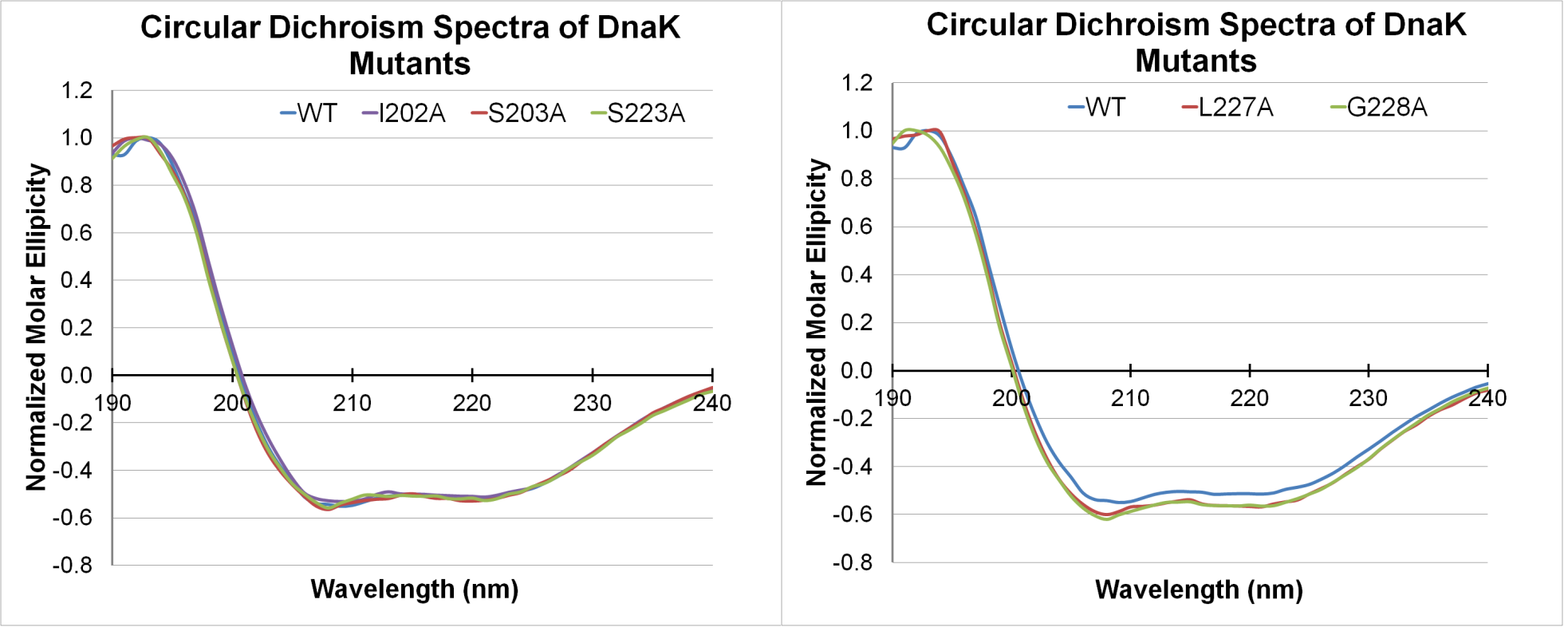

Supplement: Figure S4 — Normalized circular dichroism spectra of wild-type and mutant DnaK. All mutants have very similar fold as the wild-type DnaK. (TIF) [file pcbi.1003279.s004.tif]
