# Supplementary material for: Identification of Key Hinge Residues Important for Nucleotide-Dependent Allostery in E. coli Hsp70/DnaK
Source: PLoS Comput Biol. 2013 Nov 21;9(11):e1003279. doi: 10.1371/journal.pcbi.1003279 (PMC3836694; doi:10.1371/journal.pcbi.1003279)
Supplement: Text S1 — Primers for DnaK mutagenesis. The detailed genetic codes used for the DnaK mutants are given. (DOCX) [file pcbi.1003279.s005.docx]

**Identification of Key Hinge Residues Important for Nucleotide-Dependent Allostery**

**in *E. coli* Hsp70/DnaK**

Peter Man-Un Ung, Andrea D. Thompson, Lyra Chang,

Jason E. Gestwicki, and Heather A. Carlson

**Supporting Information**

Primers for DnaK mutagenesis.

STOP384 to Ala (TAA to GCA)

5’ - GAC AAA AAA **GCA** AAG CTT GCG GCC GCA CTC G-- – 3’

5’ - -GC GGC CGC AAG CTT **TGC** TTT TTT GTC TTT GAC – 3’

Ile202 to Ala (ATT to GCT)

5’ – -CT TTC GAT **GCT** TCT ATT ATC GAA ATC GAC GAA GTT GAC G-- – 3’

5’ – --C GAT TTC GAT AAT AGA **AGC** ATC GAA AGT ACC ACC ACC CAG GTC – 3’

Ser203 to Ala (TCT to GCT)

5’ – --C GAT ATT **GCT** ATT ATC GAA ATC GAC GAA GTT GAC GGC G-- – 3’

5’ – --C GTC GAT TTC GAT AAT **AGC** AAT ATC GAA AGT ACC ACC ACC – 3’

Gly223 to Ala (GGT to GCT)

5’ – GTT CTG GCA ACC AAC **GCT** GAT ACC CAC CTG G-- – 3’

5’ – --C CAG GTG GGT ATC **AGC** GTT GGT TGC CAG AAC – 3’

Leu227 to Ala (CTG to GCG)

5’ – ACC AAC GGT GAT ACC CAC **GCG** GGT GGT GAA GAC TTC – 3’

5’ – GAA GTC TTC ACC ACC **CGC** GTG GGT ATC ACC GTT GGT – 3’

Gly228 to Ala (GGT to GCT)

5’ – GGT GAT ACC CAC CTG **GCT** GGT GAA GAC TTC G-- – 3’

5’ – --C GAA GTC TTC ACC **AGC** CAG GTG GGT ATC ACC – 3’
